# The Gut Microbiota of Farmed and Wild Brook Trout (*Salvelinus fontinalis*): Evaluation of Feed-Related Differences Using 16S rRNA Gene Metabarcoding

**DOI:** 10.3390/microorganisms11071636

**Published:** 2023-06-22

**Authors:** Davide Mugetti, Paolo Pastorino, Chiara Beltramo, Tania Audino, Alessandra Arillo, Giuseppe Esposito, Marino Prearo, Marco Bertoli, Elisabetta Pizzul, Elena Bozzetta, Pier Luigi Acutis, Simone Peletto

**Affiliations:** 1 Istituto Zooprofilattico Sperimentale del Piemonte, Liguria e Valle d’Aosta, Via Bologna 148, 10154 Torino, Italy; davide.mugetti@izsto.it (D.M.); beltramochiara81@gmail.com (C.B.); tania.audino@izsto.it (T.A.); alessandra.arillo@izsto.it (A.A.); giuseppe.esposito@izsto.it (G.E.); marino.prearo@izsto.it (M.P.); elena.bozzetta@izsto.it (E.B.); pierluigi.acutis@izsto.it (P.L.A.); simone.peletto@izsto.it (S.P.); 2 Centro di Referenza Regionale per la Biodiversità degli Ambienti Acquatici (BioAqua), Via L. Maritano 22, 10051 Avigliana, Italy; 3 Dipartimento Scienze della Vita, Università degli Studi di Trieste, Via Giorgieri 10, 34127 Trieste, Italy; marbertoli@units.it (M.B.); pizzul@units.it (E.P.)

**Keywords:** intestinal microbiota, salmonids, brook trout, next generation sequencing, 16S rRNA

## Abstract

The gut microbiota has become a topic of increasing importance in various fields, including aquaculture. Several fish species have been the subject of investigations concerning the intestinal microbiota, which have compared different variables, including the intestinal portions, the environment, and diet. In this study, the microbiota of farmed and wild brook trout (*Salvelinus fontinalis*) were analyzed, in which the wall and content of the medial portion of the intestine were considered separately. A total of 66 fish (age class 2+) were sampled, of which 46 were wild and 20 were farmed brook trout, in two different years. Microbiota data were obtained using a 16S metabarcoding approach by analyzing the V3–V4 hypervariable regions of the corresponding 16S rRNA. The data showed that the core microbiota of these species consist of *Proteobacteria* (*Alpha*- and *Gammaproteobacteria*), *Actinobacteria*, *Firmicutes* (*Bacilli* and *Clostridia*), and, only for farmed animals, *Fusobacteria*. The latter taxon’s presence is likely related to the fishmeal-based diet administered to farmed brook trout. Indeed, alpha and beta diversity analysis showed differences between wild and farmed fish. Finally, statistically significant differences in the microbiota composition were observed between the intestinal walls and contents of wild fish, while no differences were detected in reared animals. Our work represents the first study on the intestinal microbiota of brook trout with respect to both farmed and wild specimens. Future studies might focus on the comparison of our data with those pertaining to other fish species and on the study of other portions of the brook trout intestine.

## 1. Introduction

The term “microbiota” refers to the microbial population that colonizes a certain body district or environment, while “microbiome” refers to the genetic heritage of a specific microbiota [1]. One of the most studied microbiotas is that of the gut [2,3]. The intestinal microbiota of different animal species has also been studied, especially with respect to mammals. An example is the work conducted by de Jonge and collaborators [4], who analyzed the microbiotas of 54 different mammals, clustering the data based on the different dietary habits and intestinal morphologies of the investigated species. Other studies have focused on species of zootechnical interest, such as cattle [5,6], pigs [7,8], horses [9], sheep [10], and goats [11]. 

Given the growing importance of aquaculture to the production of animal origin proteins, several researchers have also begun to study the gut microbiota of aquatic organisms. Therefore, data on intestinal microbiota of economically important fish species have been made available, including those on Nile tilapia (*Oreochromis niloticus*) [12], turbot (*Scophthalmus maximus*) [13], rainbow trout (*Oncorhynchus mykiss*) [14], Chinook salmon (*O. tshawytscha*) [15], Atlantic salmon (*Salmo salar*) [16], and common carp (*Cyprinus carpio*) [17]. Compared to mammals, the study of the intestinal microbiota of fish is more complex, as there are a greater number of variables that can influence its composition. In fact, in addition to the factors that have been extensively studied in mammals (e.g., species, diet, and age), the aquatic environment plays a preponderant role in the composition of the microbial community constituting the fish gut microbiota [18,19,20]. Despite these difficulties, the study of the intestinal microbiota of new fish species is desirable in order to increase the available knowledge regarding the composition and the influence of its modifying factors. 

In this respect, our study aimed to characterize the intestinal core microbiota of the brook trout (*Salvelinus fontinalis*), which is a fish that can be found in Italy in both a farmed form and in the environment as an invasive species [21]. This salmonid is a species of economic and environmental interest for several countries, including Italy. Regarding human consumption farming, the Italian data for 2021 reported a production of 850 tons, which is equivalent to a monetary value of 3.65 million euros [22]. Brook trout are farmed for both food and recreational fishing. This latter purpose leads to the release of brook trout in the natural environment as invasive species, which has an adverse ecological impact on the local ecosystem and fauna. Several studies have been conducted on this fish species concerning such topics as distribution [23], production performance [24], and sanitary conditions [25,26]. Although several aspects have been investigated with respect to this species, to the best of our knowledge, there are currently no studies concerning the intestinal microbiota. Therefore, our work aimed to study the microbiota of *S. fontinalis* through next-generation sequencing (NGS) 16S metabarcoding and concerning several variables, namely, different sampling sites (the natural environment and farms) and matrices (contents and intestinal walls). Moreover, we selected wild and farmed fish to investigate the influence of environmental factors, primarily diet, on microbiota composition.

## 2. Materials and Methods

### 2.1. Fish Sampling

A total of 66 brook trout (46 wild fish and 20 farmed specimens) belonging to the age class 2+ were sampled in the Piedmont region (north-western Italy). 

These 46 wild fishes were sampled from Balma Lakes during the summer of 2019 and 2020. Specifically, n = 21 and n = 25 specimens were captured on 4 August 2019 and 29 July 2020, respectively. Balma Lakes (Upper and Lower Balma) is located in the Cottian Alps at 2.101 m a.s.l. (lower Lake Balma; 45°02′13.799″ N; 07°10′52″ E) and 2.213 m a.s.l. (upper Lake Balma; 45°02′15.055″N; 07°10′27.724″ E). The lakes fall within the SAC IT1110006 Orsiera Rocciavré (Municipality of Coazze, Province of Turin, northwest Italy). The Upper Lake is S-shaped, with two sub-basins separated by a shallow mid-section. The lake’s perimeter is 774 m, with a 1.82 ha surface area and 2.77 m maximum depth. The lake is placed in a catchment core composed of ophiolite metamorphic bedrock, and the landscape is dominated by rocky outcrops, ridges, and mountain walls. The Lower Lake is circular with a perimeter equal to 414 m; its surface area is 1.21 ha, and its maximum depth is 6.42 m. The main catchment core has the same composition as that described for the upper lake, and the landscape is dominated by the same elements noted above, except for a meadow, which is absent near the Lower Lake. A small inlet, located on the western shore, divides into three small branches before entering the lake. Although a true outlet is not evident, Balma Creek originates from water filtration through the sediments at the eastern side of the basin. The most relevant anthropogenic impacts on the Balma Lakes area over the last four decades of the 20th century have been the long-distance airborne transport of pollutants from urban areas in the plains, grazing activities, and fishing. Although Balma Lakes was originally fishless, *Salvelinus fontinalis* was introduced for recreational fishing in the 1970s [21]. Fish sampling was performed according to the standardized method for fish sampling in European lakes (EN 14757:2005), which requires a single session using both benthic and mesopelagic nets corresponding to the type of the lake, its depth, and its surface. The benthic nets (length—30 m; height—1,5 m; total area—45 m^2^) were composed of 12 panels (length: 2,5 m) with a mesh size ranging from 5 to 55 mm. The mesopelagic nets (length: 27,5 m; height: 6 m; total area: 165 m^2^) had one fewer panel than the benthic nets (eleven panels in total). The nets were placed according to the bathymetric profile of the lake at approximately 6 p.m. and recovered 12 h later. Only fish belonging to the age class 2+ were selected and retrieved for the analyses. 

Regarding the farmed samples, 20 brook trout (age 2+) were sampled in October 2020 on a farm housing both this species and brown trout (*Salmo trutta*). The farm is located in a mountainous area (Cottian Alps) at about 900 m a.s.l. The water supply constituted creek water (12 °C). Salmonids were reared at low density (25 kg/m^3^) and fed twice a day with commercial feed pellets (Premium, Skretting). Brook trout were captured using a landing net and euthanized via an overdose (170 mg kg^−1^) of tricaine methanesulfonate (MS-222).

The study protocol was designed according to the guidelines of European Union Council 2010/63/EU for the use and care of experimental animals and the ARRIVE guidelines, and the principle of the 3Rs was applied.

Then, the middle intestinal tract was harvested directly on the sampling site using sterile scalpels and forceps. Gut contents were also collected by applying slight pressure on the intestinal wall to allow the contents to eject. Samples of intestinal walls and contents were transported to the laboratory under refrigerated conditions and stored at −80 °C before further analysis.

Water temperature (°C), pH (unit of pH), conductivity (µS cm^−1^), and dissolved oxygen (mg L^−1^) content were measured during fish sampling using portable probes (HI 9033 conductivity meter, HI 9125 pH/ORP meter, and HI 9147 dissolved oxygen meter, Hanna Instruments Inc., Woonsocket, RI, USA). Three replicate measurements were taken for each parameter.

### 2.2. DNA Extraction

For DNA extraction, the QIAamp^®^ PowerFecal^®^ Pro DNA Kit (Qiagen, Hilden, Germany) was used according to the manufacturer’s instructions. A portion of 50 mg of each sample was transferred to homogenization tubes containing ceramic beads with 800 µL of CD1 lysis buffer and were subjected to homogenization using the MP Biomedicals^™^ FastPrep-24^™^ Classic Bead Beating Grinder and Lysis System (Fisher Scientific Italia, Rodano, Italy), employing 1 cycle of 40 s at a speed of 10. A positive extraction control, the ZymoBIOMICS Microbial Community Standards (Zymo Research, Tustin, CA, USA), and a negative extraction control (ultrapure water) were established. The extracted DNA was immediately quantified using a VivaSpec spectrophotometer (Sartorius Stedim Biotech, Göttingen, Germany) and Qubit^™^ 3 Fluorometer (ThermoFisher, Waltham, MA, USA) via the dsDNA HS Assay Kit (ThermoFisher, MA, USA) and then stored at −20 °C.

### 2.3. 16S Ribosomal RNA (16S rRNA) Gene Metabarcoding

The samples were amplified using the 16S Metagenomic Sequencing Library Preparation protocol (Illumina, San Diego, CA, USA). The primers 341FB (5′-CCTACGGGNGGCWGCAG-3′) and 806RB (5′-GACTACHVGGGTATCTAATCC-3′) targeting the hypervariable V3–V4 regions of the 16S rRNA gene were used according to the manufacturer’s protocol. Amplicon PCR (final volume: 25 µL) was set up using 12,5 µL of NEBNext^®^ Q5^®^ Hot Start HiFi2X Master Mix (New England BioLabs, Rowley, MA, USA), 1,25 µL of each 10 µM primer, and 10 ng of DNA. The thermal profile was as follows: 98 °C × 30 s; 40 cycles at 98 °C × 10 s, 55 °C × 30 s, and 72 °C × 30 s; and final extension at 72 °C × 2 m. The PCR products were visualized on a 2% agarose gel to verify the successful amplification of the target. Samples with the amplicon of interest (430 bp) were purified using magnetic beads Agencourt AMPure XP (Beckman Coulter, Brea, MA, USA). 

Index PCR was performed using the Nextera XT Index Kit v2 Set A (Illumina, CA, USA). Specifically, 25 µL of NEBNext^®^ Q5^®^ Hot Start HiFi2X Master Mix (New England BioLabs, MA, USA), 5 µL of Nextera XT Index Primers (Primer 1 and 2), 10 µL of H20, and 5 µL of purified DNA were added. The thermal profile used was set thusly: 98 °C × 30 s; 12 cycles at 98 °C × 10 s, 55 °C × 30 s, and 72 °C × 30 s; and final extension at 72 °C × 2 m. After purification using magnetic beads Agencourt AMPure XP (Beckman Coulter, CA, USA), quality control of the purified libraries was performed using the dsDNA HS Assay Kit (ThermoFisher, MA, USA) applied to a Qubit^™^ 3 Fluorometer (ThermoFisher, MA, USA) and using the Agilent High Sensitivity DNA kit (Agilent Technologies, Santa Clara, CA, USA) applied to the BioAnalyzer 2100 Instrument (Agilent Technologies, CA, USA). The libraries were then normalized and pooled before being quantified using the NEBNext^®^ Library Quant Kit for Illumina (New England BioLabs, MA, USA). Finally, pooled libraries were normalized to 4 nM and subjected to 2 × 300 paired-end sequencing via a MiSeq^™^ System (Illumina, CA, USA) with the MiSeq Reagent Kit v3 (Illumina, CA, USA). 

### 2.4. Bioinformatics

The fastq data were analyzed using the CLC Genomics Workbench (Qiagen, Germany) software v.23, for which specific tools were used for the analysis of Operational Taxonomic Units (OTU) clustering contained in the CLC Microbial Genomics Module. Briefly, reads were filtered according to their quality scores (Qscore < 0,05), ambiguity for up to 2 nucleotides, adapter sequence cut-offs, and minimum length (minimum 100 nucleotides). Consensus sequences with forward and reverse sequences were created and submitted to the SILVA database (version 138) for OTU classification. 

Alpha diversity was estimated using bias-corrected Chao1 (yielding total species richness), Simpson index (revealing the probability that two randomly chosen individuals belong to different species), and Shannon entropy (the degree of average uncertainty relating to the classification of an unknown individual). Beta diversity was estimated using the Bray–Curtis method and principal coordinate analysis (PCoA). Since the null hypothesis for the homogeneity of variance and/or for normal distribution could not be rejected, difference in Alpha diversity among groups was analyzed using the nonparametric Kruskal–Wallis test (and the relative Mann–Whitney U post-hoc test), whereas difference in beta diversity was assessed using the PERMANOVA test. Statistical significance was set at *p* < 0.05. 

## 3. Results

### 3.1. Environmental Variables

In 2019, the mean water temperature of Balma Lakes ranged from 13.60 ± 0.52 °C (Lower Lake) to 14.10 ± 0.35 °C (Upper Lake). The pH values ranged from 6.98 ± 0.12 (Lower Lake) to 7.15 ± 0.21 (Upper Lake). The conductivity was very low and ranged from 17 ± 0.87 µS cm^−1^ (Lower Lake) to 20 ± 0.87 µS cm^−1^ (Upper Lake). Oxygenation ranged from 8.10 ± 0.68 mg L^−1^ (Lower Lake) to 8.70 ± 0.57 mg L^−1^ (Upper Lake). 

In 2020, the mean water temperature of Balma Lake ranged from 14.40 ± 0.61 °C (Lower Lake) to 15.21 ± 0.35 °C (Upper Lake). pH values ranged from 7.16 ± 0.10 (Lower Lake) to 7.31 ± 0.12 (Upper Lake). The conductivity ranged from 18 ± 0.17 µS cm^−1^ (Lower Lake) to 19 ± 0.97 µS cm^−1^ (Upper Lake). Oxygenation ranged from 7.98 ± 0.15 mg L^−1^ (Lower Lake) to 8.41 ± 0.68 mg L^−1^ (Upper Lake). 

The main physicochemical water parameters of the fish farm were as follows: water temperature: 14.21 ± 0.89 °C; pH: 7.10 ± 0.54; conductivity: 102 ± 1.12 µS cm^−1^; dissolved oxygen: 7.95 ± 0.99 mg L^−1^

### 3.2. Sequences Analyses

Following the selection of the sequences according to the established parameters, a total of 24,799,141 reads were obtained for the intestinal contents samples, while 22,108,700 were obtained for the wall samples. The SILVA database sequences were compared to 1,672,557 unique non-chimeric sequences from the intestinal contents samples and 1,203,619 from the wall samples, leading to the assignment of 2503 and 1220 OTUs, respectively. The data from the positive control corresponded with the manufacturer’s indications for the 16S sequencing protocol (all the bacterial taxa of the standard were detected in the correct percentages, whilst *Saccharomyces cerevisiae* and *Cryptococcus neoformans* were absent, as expected), while the negative extraction and PCR controls did not show any contamination.

**Figure 1:**
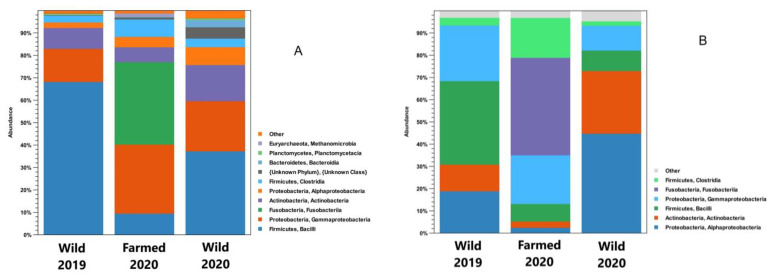
Class-grouped OTU abundance (%), with indications regarding phyla, of intestinal contents (**A**) and intestinal wall (**B**) samples in wild and farmed brook trout.

Regarding the phyla, the analysis of the obtained sequences showed that *Firmicutes*, *Proteobacteria*, and *Actinobacteria* were the most abundant, representing about 90% of the microorganisms. When the sequences were analyzed at the class level, *Bacilli*, *Gammaproteobacteria*, *Actinobacteria*, *Alphaproteobacteria*, and *Clostridia* were the most represented, constituting over 80% of the samples’ microbiota (Figure 1). Given their presence in most of the samples analyzed, these bacterial taxa can be considered the core microbiota of the brook trout midgut in both the contents and the walls. Moreover, exclusively for farmed fishes, the phylum *Fusobacteria* must be considered as part of their core microbiota. 

### 3.3. Differences between Wild and Farmed Samples: Composition of the Microbiota

Considering the data concerning the microbiota in the intestinal contents, the abundance levels of *Firmicutes* were about 71.0% (2019) and 41.0% (2020) in the wild samples, while reaching 18.0% in the farmed animals. Focusing on the samples taken from wild brook trout, *Proteobacteria* (*Alpha*- and *Gammaproteobacteria*) were the second most represented class (18.0% in 2019; 35.0% in 2020), followed by *Actinobacteria* (6.8% in 2019; 17.0% in 2020). Alternatively, the analysis of the intestinal contents of the farmed fishes showed the presence of *Fusobacteria* (37.0%), which were not detected in the samples from the natural environment. This high presence of *Fusobacteria* can be almost entirely attributed to the genus *Cetobacterium*. Focusing on *Proteobacteria*, higher percentages of *Gammaproteobacteria* were observed compared to *Alphaproteobacteria* in all the analyzed samples (both farmed and wild). Substantial differences were observed regarding the *Bacilli* class, for which the values ranged from 9.5% for farmed fish to 68.0% for wild brook trout sampled in 2019. Fewer differences were observed for the other *Firmicutes* class analyzed (*Clostridia*), whose highest percentages (7.8%) were observed in the farmed fish. 

The analysis of the intestinal walls allowed for the further observation of the differences between the farmed and wild fishes. The farmed specimens showed the presence of *Proteobacteria* (24.0%), *Firmicutes* (41.0%), and *Actinobacteria* (2.9%) in 2019, while in 2020, the same situation was seen with different proportions (56.0% *Proteobacteria*, 11.0% *Firmicutes*, and 29.0% *Actinobacteria*). In the farmed samples, a high percentage (44.0%) of *Fusobacteria* (genus *Cetobacterium*) was again observed, which were lacking in the specimens collected in the natural environment. A class-level analysis of the gut wall microbiota showed differences compared to those identified from the intestinal contents. Specifically, the wild fish sampled in 2019 showed the same wall-related trend regarding *Alpha*-, *Gammaproteobacteria*, and *Bacilli* distributions, albeit with different percentages. Alternatively, wild samples from 2020 showed a higher percentage of *Alphaproteobacteria* (45.0%) than *Gammaproteobacteria* (11.0%). 

### 3.4. Differences between Wild and Farmed Samples: Alpha and Beta Diversity

**Figure 2:**
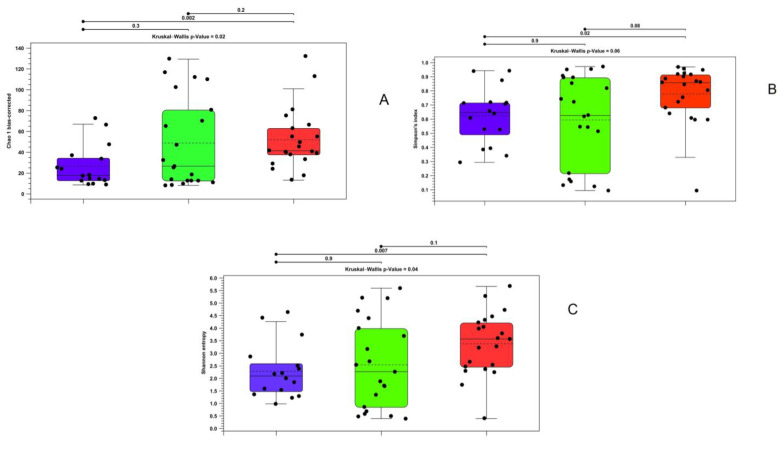
Alpha diversity calculated with bias-corrected Chao 1 index (**A**), Shannon entropy (**B**), and Simpson index (**C**) regarding gut contents samples in wild and farmed brook trout. Purple—farmed samples; green—wild samples in 2019; red—wild samples in 2020.

Alpha diversity analyses revealed significantly different richness and diversity in the intestinal contents of the analyzed samples. Particularly, the Kruskal–Wallis test yielded significant results for all the indices except the Simpson index, while the between-group Mann–Whitney U test confirmed significance between the farmed and wild brook trout in 2020 (Figure 2). 

**Figure 3:**
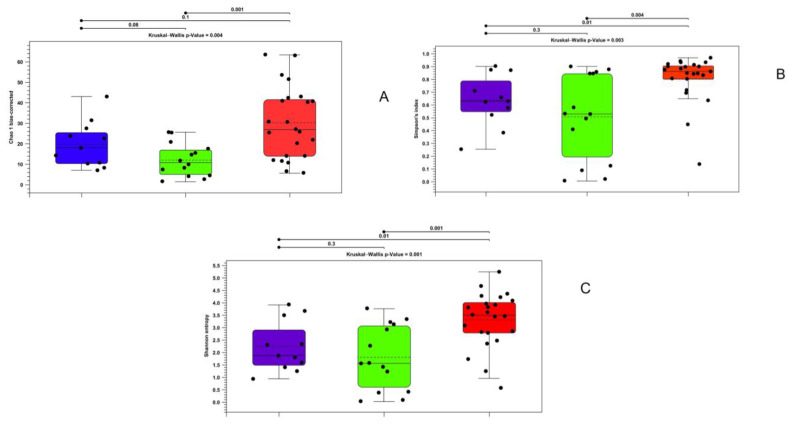
Alpha diversity calculated with bias-corrected Chao 1 index (**A**), Shannon entropy (**B**), and Simpson index (**C**) for gut wall samples in wild and farmed brook trout. Purple—farmed samples; green—wild samples of 2019; red—wild samples of 2020.

Similar results were obtained through an analysis of the data obtained from the gut walls via the Kruskal–Wallis test, which showed significant differences for all indices. For the Mann–Whitney test, only the Simpson index and Shannon entropy showed that there were statistically significant differences between the farmed and wild specimens in 2020. However, all the indices showed significant variations between the wild animals taken in the two different years (Figure 3).

**Figure 4:**
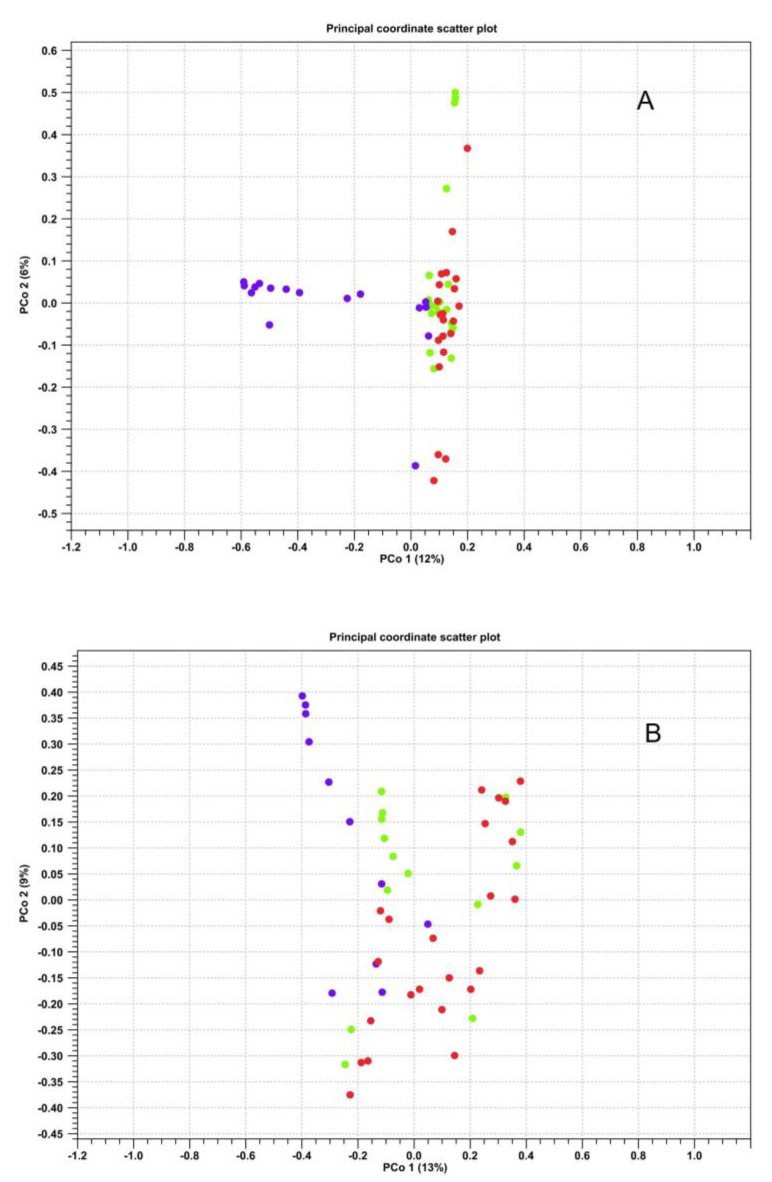
Scatter plot representing the beta diversity calculated via the Bray–Curtis index for the samples of intestinal contents (**A**) and intestinal walls (**B**). Violet—farmed samples; green—wild samples of 2019; red—wild samples of 2020.

Regarding beta diversity, statistically significant differences were observed between the farmed and wild microbiotas, especially in the case of intestinal contents. The PERMANOVA analysis also demonstrated significant differences (p Bonferroni ≤ 0.05) between the three compared groups with respect to both intestinal contents and walls (Figure 4).

### 3.5. Differences between Intestinal Walls and Contents: Composition of the Microbiota

Besides the comparison between wild and farmed fish, the microbiota of the two analyzed matrices (contents and intestinal walls) were also compared. In the farmed brook trout, the microbiota compositions at the phylum level were similar, with a very high percentage of *Fusobacteria* (37.0% in contents and 44.0% in walls) and *Proteobacteria* (25.0% and 35.0%), followed by a lower level of *Firmicutes* (18.0% and 25.0%).

**Figure 5:**
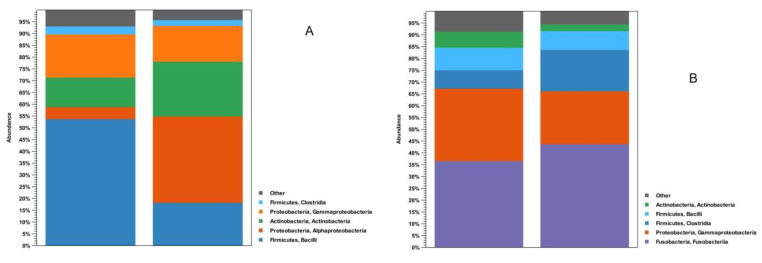
OTU abundance (%) grouped by class (with indications regarding phyla) of wild (**A**) and farmed (**B**) samples.

Conversely, differences were noted regarding the samples of wild fish, especially with respect to the percentages of *Firmicutes* and *Proteobacteria*. *Firmicutes* was prevalent in the intestinal contents (57.0% of the microbiota), while *Proteobacteria* was prevalent in the gut wall (52.0%). *Bacilli* was present at a higher percentage in the intestinal contents (54.0%) compared to the wall (18.0%). Finally, the greater prevalence of *Proteobacteria* in the gut wall was mainly due to *Alphaproteobacteria* (37.0% against 5.1% in the contents) (Figure 5).

### 3.6. Differences between Intestinal Walls and Contents: Alpha and Beta Diversity

**Figure 6:**
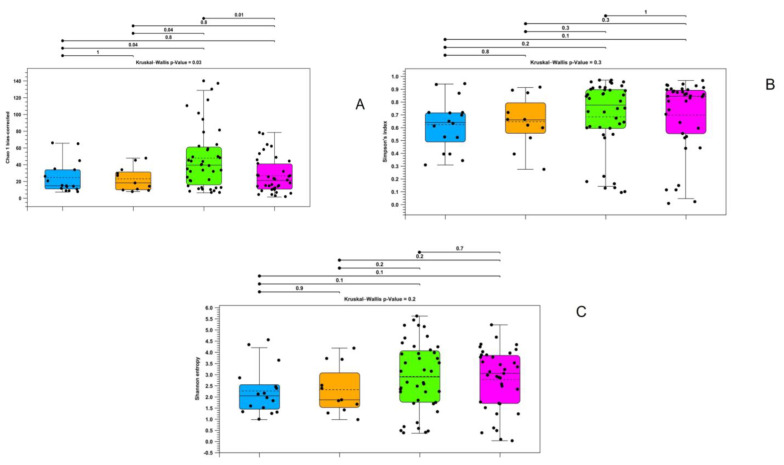
Alpha diversity calculated with bias-corrected Chao 1 index (**A**), Shannon entropy (**B**), and Simpson index (**C**) for gut wall samples in wild and farmed brook trout. Blue—gut contents of farmed samples; orange—gut wall of farmed samples; green—gut contents of wild samples; green—gut wall of wild samples.

The alpha diversity analysis did not reveal differences between the intestinal wall and contents samples (Figure 6). 

**Figure 7:**
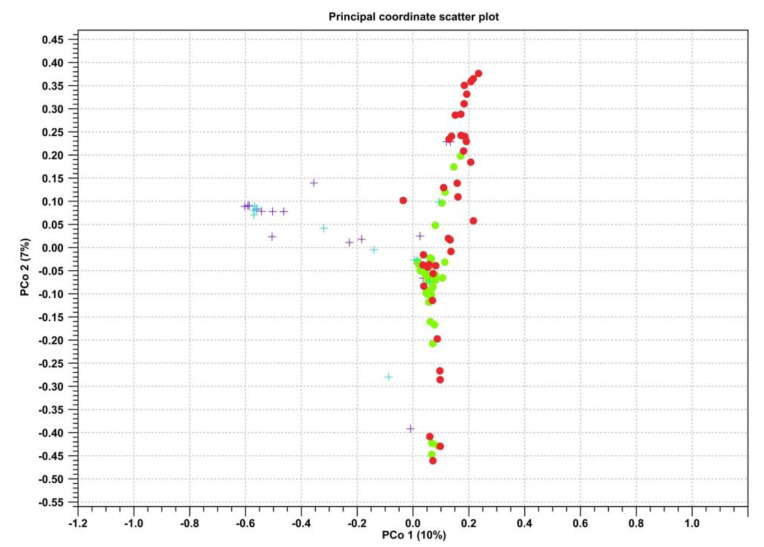
Scatter plot representing the beta diversity calculated using the Bray–Curtis index for the samples of intestinal contents (green dots) and intestinal walls (red dots) in the wild samples and intestinal contents (purple crosses) and intestinal walls (blue crosses) in the farmed samples.

In contrast, the comparison between groups with beta diversity showed differences between the two biological districts in wild fish, which were confirmed according to the significant values obtained in the PERMANOVA analysis (p Bonferroni ≤ 0.05) (Figure 7).

## 4. Discussion and Conclusions

The most represented microorganisms in the analyzed microbiotas were *Proteobacteria* (*Alpha*- and *Gammaproteobacteria*), *Actinobacteria*, *Firmicutes* (*Bacilli* and *Clostridia*), and, only in the reared specimens, *Fusobacteria*. These data are in agreement with those from previous studies. Indeed, Kim et al. [19] identified *Proteobacteria* and *Firmicutes* as the most abundant taxa in fish microbiota and reported a high percentage of *Fusobacteria* in freshwater species (especially Perciformes, Tetraodontiformes, Siluriformes, Cypriniformes, and Lophiiformes); however, they did not consider salmonid species in their study. Specific studies have been conducted on the gut microbiota of salmonids. The intestinal microbial composition of reared Atlantic salmon (*Salmo salar*) fed with fishmeal-free feed showed that *Firmicutes*, *Proteobacteria*, and *Actinobacteria* were the most represented taxa [27]. The microbiota of juvenile rainbow trout (*O. mykiss*) analyzed by Michl et al. [28] consisted mainly of the phyla *Proteobacteria*, *Firmicutes*, *Bacteroidetes*, *Fusobacteria*, and *Actinobacteria*. They also studied the variations of the intestinal microbiota of this species in relation to diet, reporting an increase in *Clostridiales* (*Firmicutes*), *Fusobacteriales* (*Fusobacteria*), *Vibrionales*, and *Alteromonadales* (*Gammaproteobacteria*) in relation to an animal-protein-rich diet. Based on these data, the classes *Alpha*- and *Gammaproteobacteria*, *Actinobacteria*, *Bacilli*, and *Clostridia* should be considered the “core” microbiota, as they are present in more than 80% of the samples. 

*Fusobacteria* were also identified in farmed samples. In the study conducted by Lyons et al. [29] on the intestinal contents of rainbow trout, a high presence of *Fusobacteria* was detected, although, in this case, the most represented class was *Mollicutes*, followed by *Bacilli*, *Clostridia*, *Gammaproteobacteria*, and *Spirochaetia*; however, it should be considered that this work focused on the medial portion of the intestine. Therefore, these data suggest that the high presence of *Fusobacteria* may be connected to an animal-protein-based diet, as underlined by other studies carried out on reared teleosts fed fishmeal feed [19,30]. The *Fusobacteria* found in our study are all attributable to the *Cetobacterium* genus. *Fusobacteriales*, especially *Cetobacterium* spp., are negatively correlated with the dietary availability of vitamin B12 (cyano-cobalamin) [31]. This vitamin is highly present in fish [32], so a diet rich in fishmeal-based feed can increase the presence of vitamin B12-synthesizing bacteria, such as *Fusobacteria*. The comparison between the intestinal microbiotas of the wild and farmed brook trout seems to support this hypothesis, as demonstrated by the high percentages of *Fusobacteria* found in specimens fed commercial feed. 

Upon analyzing the two biological matrices separately, the data from our study do not show differences between the microbiota of the intestinal walls and contents. Our results are in contrast with observations made in previous studies. Nyholm et al. [33] and Gajardo et al. [34] compared the microbiota of the intestinal wall and contents in three species of *Cyprinodontiformes* and in *S. salar*, respectively. Both showed that the bacterial community in the wall was significantly less different than the microbiota of the intestinal contents, indicating that only few bacterial taxa of the intestinal tract have the ability to colonize the host’s mucosa. Other studies considering a greater number of samples and other districts of the intestine (proximal and distal portions) may be needed to confirm our data. The alpha diversity analysis of the microbiotas of the intestinal wall and contents did not show significant differences for the farmed brook trout. However, the analysis of beta diversity showed differences for wild fish, with a greater presence of *Firmicutes* in the contents and *Proteobacteria* in the wall. Several studies have also found a high percentage of *Proteobacteria* associated with the intestinal wall, usually corresponding to 30–40% of the total microbiota [34]. The lack of this difference in farmed fish could be linked to the environmental standardized conditions. It is known that even differences in the wild environment can cause variations at the level of the microbiota. Nyholm et al. [33] showed significant differences in the intestinal microbial communities of three fish species (*Aphanius iberus*, *Gambusia holbrooki*, and *Valencia hispanica*) in relation to the sample collection sites and demonstrated that localization could explain a large part of the variance found. 

Therefore, our work represents the first study on the characterization of the intestinal microbiota of brook trout. The core microbiota was determined for both farmed and wild specimens. The decision to analyze the gut microbiota of brook trout in natural conditions and in artificial housing derived from what was previously performed by other authors for other fish species [35]. Differences were found in the composition of the microbiota of the groups taken into consideration; it remains for future studies to clarify what these differences are related to. The presence of *Fusobacteria* in the farmed specimens can be related to the commercial diet, as previously discussed, but what remains to be clarified is whether the other noted differences may be related to diet since the environmental parameters considered herein (water temperature, dissolved oxygen, conductivity, and pH) were quite similar between Balma Lake and the fish farm. Both the intestinal wall and the contents were taken into consideration, but no significant differences were noted between the two matrices as had been indicated in other studies. Differences were mainly found between the wild and farmed fish, and this in agreement with other studies carried out on this topic. Future investigations might focus on comparing the microbiota of species that are phylogenetically similar (e.g., salmonids) or farmed on the same farms (e.g., rainbow trout). Furthermore, the other gut districts can be studied to detect differences from the medial portion of the intestine. 

Moreover, regarding wild brook trout, new studies should be focused on fish living in alpine lakes at different altitudes to understand if this factor can influence the gut microbiota. Indeed, altitude could influence the diets of these fish (i.e., the presence or the absence of a particular prey due to its altitudinal range), thus introducing another variable to take into consideration. We have stated that three main groups (Diptera Chironomidae, Imenoptera, and Coleoptera) constitute the preferred diet of brook trout in Balma Lake [36]. However, we think that the difference in gut microbiota between the wild fish captured in 2019 and 2020 should be further analyzed while employing other environmental variables. 

All the main aquatic physicochemical parameters except temperature were quite similar for both the wild and farmed fish. In this regard, the differences in microbiota richness and diversity observed in the wild fish (2019 vs. 2020) could be related to the slight increase in water temperature that occurred in 2020. However, further studies are needed to better understand the influence of this key variable on fish gut microbiota.

Finally, the information derived from this study can represent a starting point for the evaluation of the effect of candidate probiotics on the prevention of infectious diseases, the modulation of the immune system, and the implementation of greater production performance [37]. Although our study does not provide information on probiotics, the knowledge it provides on the gut core microbiota of brook trout in healthy conditions could be the starting point for the application of probiotics in cases of dysbiosis caused by infectious processes. Thus, experimental studies on the evaluation of microbiota changes due to infectious processes are needed in the near future. The analysis of the microbiota relating to infectious diseases is of crucial importance for the development of intensive aquaculture, as demonstrated by the growing number of studies on the topic [38].

## Data Availability

Not applicable.
